# “As long as you learn to adapt”–a longitudinal mixed-methods study exploring the first decade with rheumatoid arthritis

**DOI:** 10.1186/s41927-025-00485-z

**Published:** 2025-03-24

**Authors:** Maria Bergström, Åsa Larsson Ranada, Annette Sverker, Ingrid Thyberg, Mathilda Björk

**Affiliations:** 1 Department of Health, Medicine and Caring Sciences, Linköping University, Norrköping, Sweden; 2 Pain and Rehabilitation Center, Department of Activity and Health, Department of Health, Medicine and Caring Sciences, Linköping University, Linköping, Sweden; 3 Department of Rheumatology in Östergötland, Department of Biomedical and Clinical Sciences, Linköping University, Linköping, Sweden; 4 Pain and Rehabilitation Centre, Department of Health, Medicine and Caring Sciences, Linköping University, Linköping, Sweden

**Keywords:** Disability, Everyday life, Mixed-methods research, Rehabilitation, Rheumatoid arthritis

## Abstract

**Background:**

Early diagnosis and modern treatment have changed everyday life of patients with rheumatoid arthritis (RA). However, symptoms are still pronounced several years after diagnosis. The aim of this study is therefore to synthesise the perception of everyday life in men and women with contemporary treated RA over the course of the first decade after diagnosis. This will be achieved by comparing subjective experiences with quantitative measures of disability and disease activity.

**Methods:**

A longitudinal convergent mixed method was used. Thirty-one patients, clinically diagnosed with RA and ≥ 18 years of age, were recruited from the TIRA-2 project in southeast Sweden. Patients were followed over a decade regarding disease activity (DAS28), grip force (Grippit), pain intensity (VAS mm) and activity limitations (HAQ). Participation in valued life activities (VLA-swe) was assessed 10 years after diagnosis. The patients took part in individual interviews three- and ten-years post-diagnosis. Quantitative data were analysed through descriptive analyses and linear mixed models. The interviews were analysed using directed content analyses. The results from the quantitative and qualitative analyses were integrated in accordance with the chosen design.

**Results:**

Discrepancies between the quantitative and qualitative results were revealed, along with differences between sexes. Women expressed more problems related to disease activity and grip force, which did not coincide with the quantitative results. In fact, women experienced difficulties in activities despite decreased disease activity. Furthermore, their pain score changed quantitatively over time, which was not expressed in the interviews. These disconfirming results were not seen in men. Both women and men displayed confirming results regarding activity limitation. Some issues, such as with basic needs, were more visible quantitatively than through interviews.

**Conclusions:**

Men and women with contemporary treated RA still experience disability a decade after diagnosis. Additionally, patients’ experiences and quantitatively measured outcomes do not always coincide. The qualitative data adds information and thereby complements the quantitative data on disability. Our results confirm the importance of person-centred rehabilitation in optimising patients’ possibilities for participation in everyday life.

**Clinical trial number:**

Not applicable.

**Supplementary Information:**

The online version contains supplementary material available at 10.1186/s41927-025-00485-z.

## Background

In rheumatoid arthritis (RA), routines for early diagnosis and early treatment have been available for decades. These include Disease-Modifying Anti-Rheumatic Drugs (DMARDs), with the goal of remission or low disease activity [1]. From a longitudinal perspective, even though the effects of early interventions show less disease activity [2], symptoms are still evident several years after diagnosis. For example, patients still experience affected grip force five years after diagnosis [3] and unacceptable pain (≥ 40 mm VAS) 15 years after diagnosis, despite remission [4]. Moreover, after an initial decrease, activity limitation is also substantial and still increasing 20 years after diagnosis [5]. Some differences between women and men have been stated. For example, women exhibit more disease activity, pain, activity limitation [6, 7], and lower grip force [3] than men. They also achieve remission less frequently than men [6, 8]. Although issues with symptoms are seen in both women and men and are consistent with patients treated in accordance with earlier procedures [9].

From a clinical perspective, the goal of remission or low disease activity can currently be considered accomplished in many patients with RA. However, patients connect remission to reduced impact on functioning as well as to the ability to perform valued activities [10]. In relation to these perceptions, even lower levels of pain have been related to difficulties in performing valued life activities [11]. Hence, symptoms affect patients’ everyday lives, such as by forcing them to adapt activities [12] and by negatively impacting social function [13–15]. To experience greater participation in, for example, social activities, current RA patients express a desire for symptom reduction [16]. However, through qualitative research, participation restrictions are proven to still be evident several years after diagnosis, affecting different aspects of everyday life [14, 15], including work [17] and intimate life [18]. In other words, previous research indicates discrepancies between accomplished clinical goals and the patients’ own experiences and wishes.

Through mixed methods research, we aim to look at today’s patients from both a qualitative and quantitative perspective, targeting these indicated discrepancies and enhancing the understanding of patients’ everyday life. Previously, mixed methods research in RA has focused on specific aspects such as types of treatment, but not on wider concepts like disability or everyday life. Furthermore, despite frequent longitudinal research in RA patients, longer follow-ups covering experiences of patients undergoing today’s contemporary treatment are lacking. Through a longitudinal mixed methods approach, we have the potential to increase knowledge of RA’s influence on patients’ participation in everyday life over a longer time period. The aim of this study is therefore to synthesise the perceptions of everyday life in men and women with contemporary treated RA by first exploring how they experience their everyday lives over the course of the first decade following diagnosis; and second, by comparing these experiences to quantitative measures of disability and disease activity during the same period.

## Methods

### Design

**Fig. 1:**
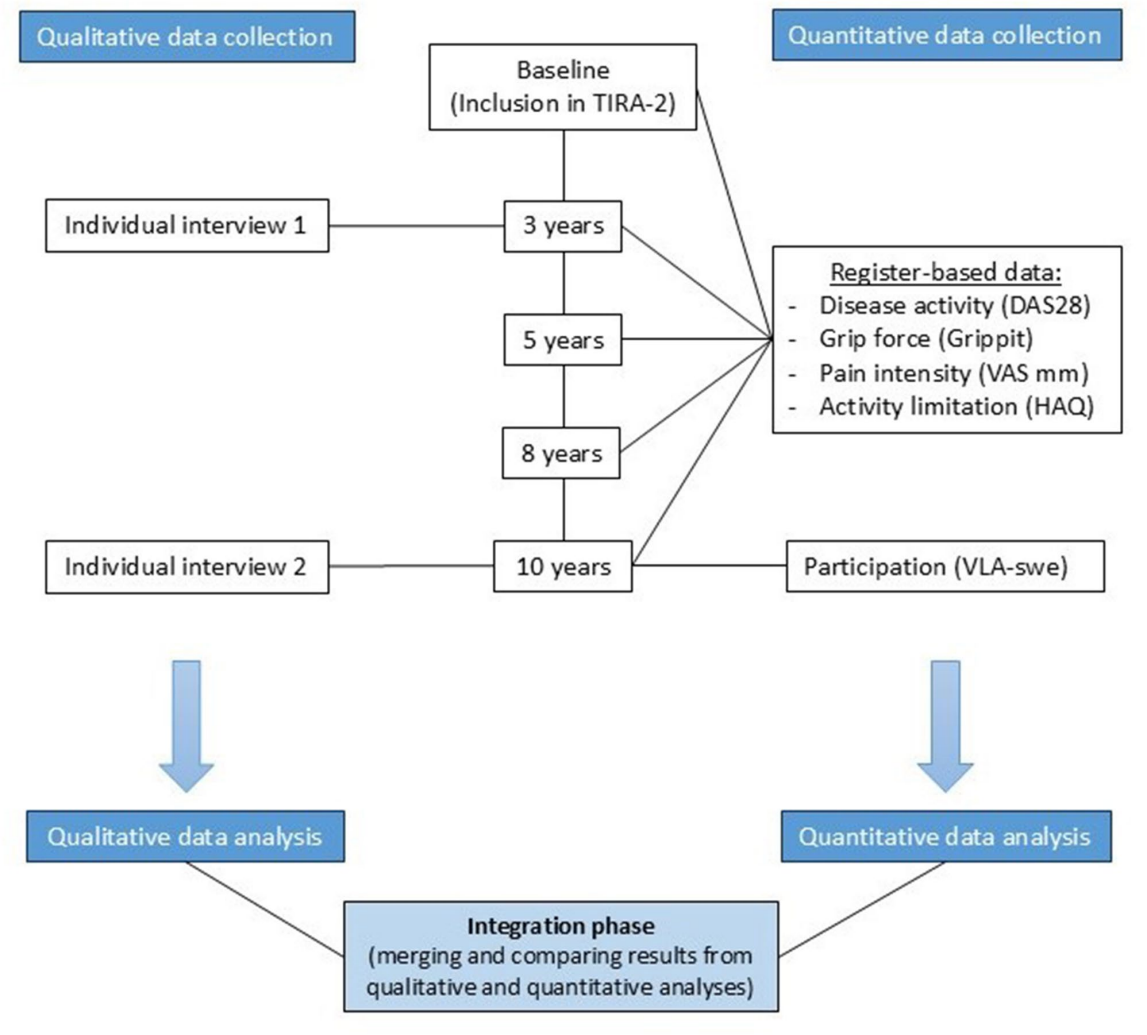
Flowchart of data collection and data analysis

To present both subjective experiences and quantitatively assessed aspects of patients’ everyday lives, this longitudinal study employed a convergent mixed methods design. This approach collects and analyses quantitative and qualitative data separately and in parallel. Afterwards, the results are integrated and compared to determine whether they confirm or disconfirm each other. The results are presented with a weaving approach [19]. The study has taken into consideration the Mixed Methods Reporting in Rehabilitation & Health Science (MMR-RHS) [20]. A flowchart of data collection and data analysis is illustrated in Fig. 1.

### Participants

This mixed methods study is part of TIRA-2 (Early Interventions in Rheumatoid Arthritis) [21], a multicentre project that consecutively included patients with RA in southeast Sweden between 2006 and 2009. Inclusion criteria for TIRA-2 were at least four of the criteria according to the American College of Rheumatology (ACR-87) [22]; or, at a minimum, morning stiffness, symmetrical arthritis and arthritis of the small joints; or a positive Anti-CCP and at least one peripheral joint with synovitis. Since inclusion in TIRA-2, these patients have been to yearly clinical follow-ups. From a total of 522 monitored patients, 59 fulfilled the criteria for and accepted participation in individual interviews during 2009–2010. Inclusion criteria for these interviews were being of working age (< 64 years of age) and having 3 years’ experience with RA. The 59 patients who took part in the first set of interviews were invited to another set of interviews in 2018–2019. Inclusion criteria for these interviews were having participated in the previous interview. Thirty-one patients accepted, and constitute the present study’s sample.

### Data collection

Quantitative register-based data and qualitative interview data were collected in parallel over 10 years. The quantitative register-based data were registered yearly in the Swedish Rheumatology Quality Register (SRQ) as part of clinical routine. Data from time for diagnosis and inclusion, as well as follow-ups at the three-, five-, eight- and ten-year marks are analysed in this study. Individual qualitative interviews were conducted approximately three and 10 years after inclusion.

#### Quantitative data collection

Disease activity was assessed using the Disease Activity Score in 28 joints (DAS28), a scale ranging from 0 to 10 with values > 5.1 indicating high disease activity, ≤ 5.1 moderate disease activity < 3.2 low disease activity, and < 2.6 remission [23]. Grip force was tested in newtons (N) using a Grippit™ (AB Detektor, Gothenburg, Sweden), using the right hand’s mean score over 10 s [24, 25]. Pain intensity was reported in millimetres (mm) on a Visual Analogue Scale (VAS), reaching between 0 and 100 mm. Activity limitation was self-reported using the Health Assessment Questionnaire (HAQ), consisting of questions regarding the ability to perform activities such as dressing, taking a tub bath, and opening car doors. For each activity, the response alternatives are “without any difficulty” (scoring 0), “with some difficulty” (scoring 1), “with much difficulty” (scoring 2), and “unable to do” (scoring 3), resulting in a total score between 0 and 3 [26].

Ten years after inclusion, patients reported their performance in valued activities using the Swedish version of the Valued Life Activity scale (VLA-swe) [27], which aims to assess participation in valued life activities. It consists of 33 activities, and is self-rated on a four-point scale (from 0 = no difficulty to 3 = unable to perform) [28]. Examples of activities include basic needs, gardening, social events, leisure activities and working. Activities not applicable or important to the individual are not included in the scoring. If an activity was reported as “unable to perform”, with “a little difficulty”, or “lot of difficulty”, it was considered “affected” in this study. VLA-swe has been tested for internal consistency and construct validity with good results [27]. Data regarding the patients’ VLA scores were collected by authors MBe and ÅLR (Fig. 1).

#### Qualitative data collection

During the first set of interviews, a semi-structured interview guide (Supplementary file 1) with open-ended questions was used, featuring questions about participation and participation restrictions in everyday life. Examples of questions were: “How is your everyday life?” and “Can you describe a situation, preferably during the past week, when you were hindered by or reminded of your RA?”. These interviews lasted between 45 and 90 min and data collection took place between October 2009 and May 2010. The second data collection period used an interview guide based on the guide from the first interviews, also featuring questions on everyday life and participation, with support as an added topic (Supplementary file 2). These interviews lasted between 14 and 109 min (median 58 min) and data were collected between October 2018 and November 2019. All interviews – both the first and second round – were audio recorded and transcribed verbatim. Prior to both sets of interviews, pilot interviews were conducted but are not part of the analysis. In total, five different researchers conducted the different sets of interviews, however, none of the researchers were involved in the patients’ treatment or rehabilitation on any occasion.

### Data analysis

Following the convergent mixed methods procedure [19], the register-based data and interview data were analysed separately. The quantitative register-based data concerning disease activity, grip force, pain, and activity limitation, as well as data on valued life activities, were analysed using IBM SPSS Statistics 28. The two sets of qualitative interview material were analysed using directed content analysis [29]. In accordance with the chosen method, a narrative integration with a weaving approach was used to compare and synthesise the results. The two types of results are presented together as a whole, category by category.

#### Statistical analysis

Descriptive statistical analyses were performed on demographical data to describe the participants, who were divided into groups based on sex. We conducted linear mixed model analysis to examine the effects of timepoint and sex on disease activity (DAS28), grip force (Grippit), pain intensity (VAS), and activity limitations (HAQ). The analysis was performed using the MIXED procedure in SPSS. The model included fixed effects for timepoint, sex, and their interaction. Restricted maximum likelihood estimation was used to fit the model, which can handle missing values in the outcome variables. The Satterthwaite approximation was applied to determine the degrees of freedom for the fixed effects. An unstructured covariance matrix was specified for the repeated measures to account for within-subject correlations. We specified several custom hypothesis tests to evaluate within-subject changes at different timepoints (three, five, eight, and 10 years) compared to baseline for both females and males. Additionally, we tested for between-subject effects to compare the differences between females and males at each timepoint. Estimated marginal means were computed for each timepoint and sex, and pairwise comparisons were performed to further explore the interaction effects. Bonferroni correction was applied to control for multiple testing.

A value of *p* < 0.05 was considered statistically significant. The results from the statistical analyses are presented in the different categories in the Results section, together with the results from the qualitative analysis, in accordance with the chosen methodological approach.

#### Qualitative analysis

A deductive directed content analysis was performed where the quantitative outcomes of disease activity, grip force, pain intensity, activity limitation, and performance in valued life activities acted as predetermined codes [29]. These codes guided the analytical process, together with the overarching question “Over the course of a decade, how is your everyday life affected by RA?” For example, since disease activity is connected to swollen joints, passages concerning problematic swollen joints were identified in the qualitative material. Similarly, activities such as getting dressed and running errands were connected to HAQ. In addition, and in relation to the overarching question, passages regarding participation and activities not related to the specific outcomes were identified. The qualitative analysis was conducted by the first author (MBe) who undertook the analysis with a manifest focus and discussed the results with co-authors. The results from the qualitative analysis are presented together with the results from the quantitative analysis in the Results section’s categories.

#### The integration phase

The results from the quantitative and qualitative analyses were merged in the integration phase with the aim to compare the quantitative outcomes with the patients’ own descriptions. In the interviews, activities were considered negatively affected if patients mentioned them as, e.g., problematic or mentioned that they withdrew from them. This integration elevated the results to two different categories. The first author (MBe) performed the integration phase, and the developed categories were discussed among co-authors until consensus was reached. In accordance with the chosen methodological approach, the results from the integration phase will be presented via a weaving approach, where categories encompassing both quantitative and qualitative data are presented one at a time [19].

## Results

**Fig. 2:**
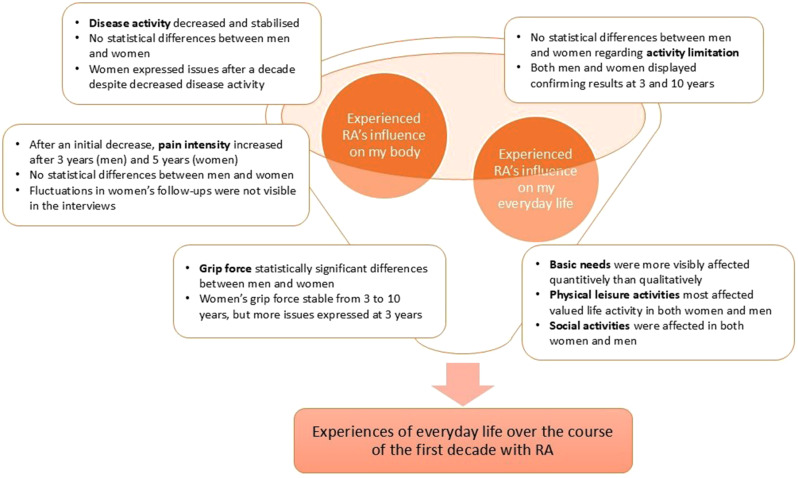
Patients’ experiences of everyday life with RA. Integration of quantitative and qualitative results of patients’ experiences of everyday life over the course of the first decade after RA diagnosis

Through the integration phase, two different categories were determined. One category – Experienced RA’s influence on my body – relates to bodily aspects such as pain and stiffness, and the other category – Experienced RA’s influence on my everyday life – relates to how RA affects activities in patients’ everyday lives during the first decade after diagnosis (Fig. 2).

### Participant characteristics

In the sample (52% women), almost all patients (97%) were prescribed DMARDs upon inclusion. Most were employed or studied at inclusion, whereas at 10 years, more had left the work force, often due to retirement. More patients also lived with other diagnoses aside from RA at the ten-year follow-up. The patients constitute a sample with a moderate disease activity, low grip force, pain intensity above an acceptable level, and moderate activity limitations (Table 1).

**Table 1:** Patient characteristics at inclusion in TIRA-2 and at the ten-year follow-up

*N*, mean(SD)		Inclusion				10 years		
	Women (*N* = 16)	Men (*N* = 15)	Total	p	Women	Men	Total	p
Age (years)	48(10)	50(10)	49(10)	0.69	-	-	-	
Educational level N Primary Secondary Higher education	4 7 5	5 8 2	9 15 7					
Co-habitating, partner N (%)	10 (63%)	11 (73%)	21 (68%)		11 (69%)	12 (80%)	23 (74%)	
Other diagnoses N (%)	1 (6%)	1 (7%)	2 (6%)		8 (50%)	3 (20%)	11 (35%)	
Employed, student N (%)	15 (94%)	12 (80%)	27 (87%)		6 (38%)	10 (67%)	16 (52%)	
DMARDs N (%)	16 (100%)	14 (93%)	30 (97%)		8 (50%) ^1^	11 (73%) ^2^	19 (61%) ^3^	
DAS28 score	5.1(1.3)	4.4(1.2)	4.8(1.3)	0.090	2.6(1.2)	2.3(0.9)	2.5(1.1)	0.967
Grippit Newton	134(75)	152(87)	143(81)	0.615	141(79)	344(58)	208(122)	< 0.001*
Pain intensity VAS mm	48(25)	43(23)	46(24)	0.589	32(27)	35(33)	34(30)	0.882
HAQ score	0.7(0.6)	0.9(0.5)	0.8(0.5)	0.302	0.6(0.4)	0.5(0.4)	0.5(0.4)	0.852
Number of affected VLA’s	-	-	-		7(8)	6(8)	7(8)	

N: number of patients. DMARDs: Disease-Modifying Anti-Rheumatic Drugs. DAS28: Disease Activity Score in 28 joints. VAS: Visual Analogue Scale. HAQ: Health Assessment Questionnaire. VLA: Valued Life Activities. * Statistically significant by < 0.05. ^1^ 7 (44%) unknown. ^2^ 4 (26%) unknown. ^3^ 11 (35%) unknown

### Experienced RA’s influence on my body

**Fig. 3:**
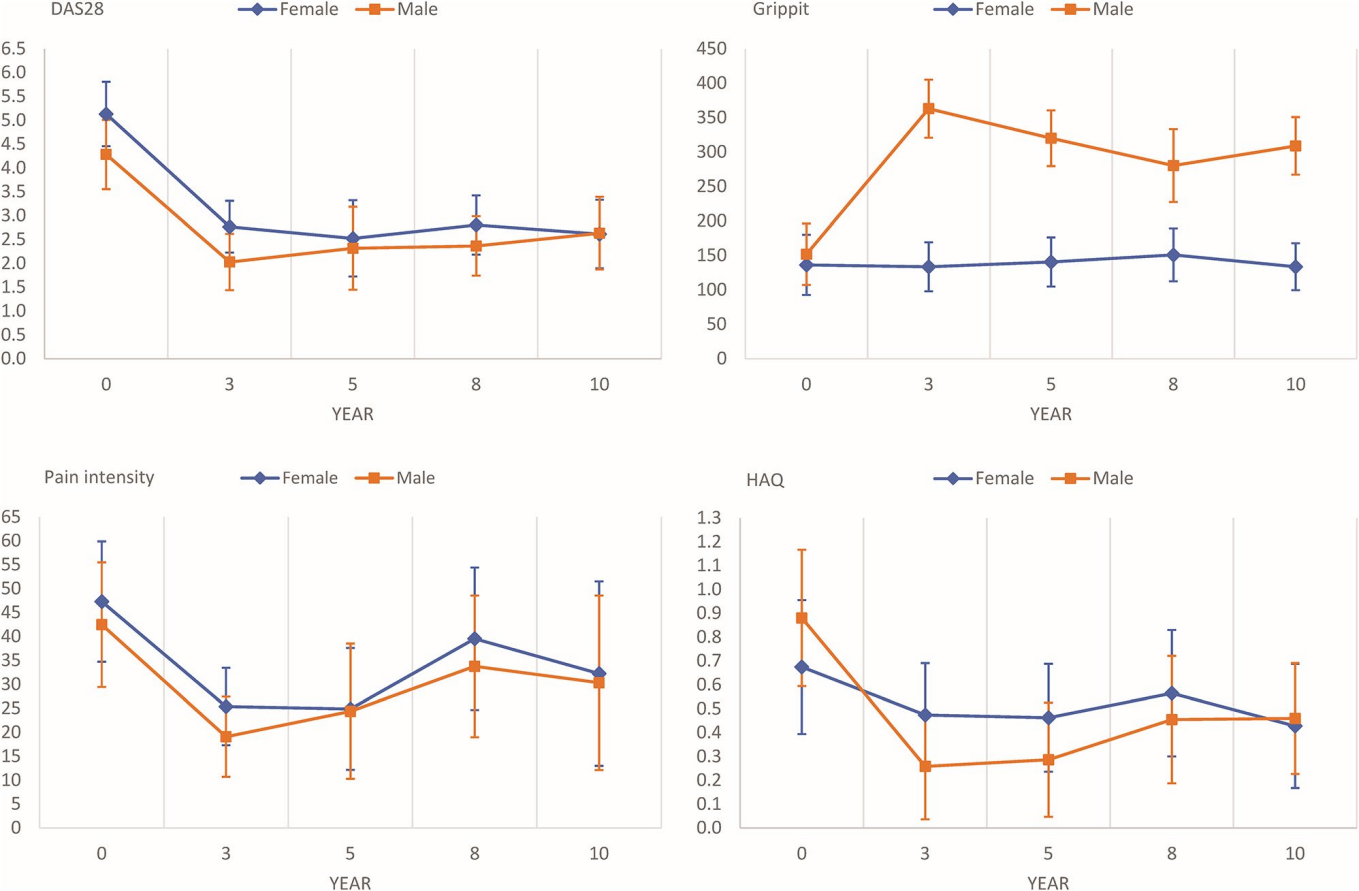
Disease activity, grip force, pain intensity, and activity limitation in patients with RA. Disease activity score 28 joint count (DAS28 score), grip force (Newton), pain intensity (VAS mm) and Health Assessment Questionnaire (HAQ), from inclusion (year 0) to 10 years in women and men with RA

The quantitative analysis showed that disease activity and pain intensity decreased in both men and women from inclusion on. However, while disease activity stabilised after three years, pain intensity increased in men after three years (mean 19, 95% CI = 10 to 27 at three years, mean 24, 95% CI = 10 to 39 at five years) and in women after five years (mean 25, 95% CI = 12 to 38 at five years, mean 40, 95% CI = 25 to 54 at eight years) (Fig. 3). Regarding disease activity and pain intensity, there were no statistical differences between women and men during the first decade with RA, although women generally displayed higher values.

In relation to disease activity, stiffness was expressed as problematic, such as after sitting down or getting up from the floor. After a decade, both men and women still mentioned stiffness, as exemplified by one woman:

*…I used to say that it is like the hinges need lubrication in the knees. (Woman*,* age 68*,* 10 years after diagnosis)*

Flares were expressed by both men and women and both after three and 10 years. In connection to flares, issues with pain, swelling, stress, and fatigue were mentioned. Patients could be highly affected during flares, such as exemplified by one woman:

*When I am in these flares*,* then I absolutely feel it*,* then the body is on*,* it is*,* it is like turning on a refrigerator*,* this buzzing you hear in the entire body*,* when you feel the whole body when it is on*,* so to speak … (Woman*,* age 55*,* three years after diagnosis)*.

The quantitative analysis showed that disease activity according to DAS28 increased in men between three (mean 2.0, 95% C = 1.4 to 2.6) and 10 years (mean 2.6, 95% CI = 1.9 to 3.4), which is also seen in the men’s descriptions in the qualitative analysis, where they expressed issues to a larger extent 10 years after diagnosis, namely, nine men mentioned specific issues at three years, compared to that thirteen mentioned issues at 10 years. This illustrates *confirming* results. On the other hand, disease activity decreased slightly, although not significantly, in women between three (mean 2.8, 95% CI = 2.2 to 3.3) and 10 years (mean 2.6 95% CI = 1.9 to 3.4), whereas problems with stiffness and flares were still mentioned a decade after diagnosis (six women described issues at three years, 12 women described issues at 10 years) In contrast to men, women exhibit *disconfirming* results regarding disease activity.

Pain was connected to stiffness and often to the joints. Men in particular described pain as constant both at three and 10 years. Pain was inevitably a factor that affected the patients, for example, by causing disturbed sleep. Generally, morning was pronounced as more difficult, regarding stiffness and pain, and in both women and men. For example:

*…yesterday morning it hurt*,* really and stiff*,* so*,* and then off course*,* but I do what I should anyway even though it hurts. (Man*,* age 54*,* three years after diagnosis)*.

As seen in the quantitative analysis, pain intensity increased in men between three (mean 19, 95% CI = 10 to 27) and 10 years (mean 30, 95% CI = 12 to 49) in men. This mirrors the results from the qualitative analysis, where men expressed issues with pain to a larger extent at 10 years after diagnosis (11 described problems at three years, 13 described problems at 10 years). Conversely, in women, pain was expressed in similar ways at both three (12 mentioned problems) and 10 years (13 mentioned problems) in the interviews, despite fluctuations seen in the quantitative data (mean 25, 95% CI = 17 to 33 at three years, mean 40, 95% CI = 25 to 54 at eight years and, mean 32, 95% CI = 13 to 52 at 10 years) (Fig. 3).

Connected to stiffness, the patients described feeling clumsy and fumbling, and how it could cause problems with gripping and pinching. Both men and women described their hands getting weaker, both at three and 10 years after diagnosis. Apart from two men, who experienced an improved condition. These, in most cases, declining experiences, impacted the patients’ lives in several respects. Regarding grip force, there were statistically significant differences between men and women at three, five, eight, and 10 years (all *p* < 0.001). After scoring more similarly at inclusion, men consistently scored higher in grip force, as measured by Grippit. Generally, men expressed issues to a larger extent 10 years after diagnosis (mentioned by 11), compared to three years after diagnosis (issues mentioned by eight). This is in line with the quantitative outcomes demonstrating mean scores of 363 N (95% CI = 321 to 405) at three years and 309 N (95% CI = 267 to 351) at 10 years. Women quantitively exhibited a more stable progress regarding grip force during the first decade after diagnosis, scoring very similar at three (Newton mean 134, 95% CI = 98 to 169) and 10 years (Newton mean 134, 95% CI = 100 to 168) after diagnosis. However, women articulated more problems in relation to grip force – such as lifting heavy objects – three years after diagnosis (15 mentioned at three years, 11 mentioned at 10 years).

In summary, in men, the quantitative and qualitative analyses revealed *confirming* results regarding disease activity, pain, and grip force over the first decade after RA diagnosis. Whereas in women, the analyses showed *disconfirming* results regarding all these three bodily aspects. For example, women still expressed issues in relation to disease activity a decade after diagnosis, despite decreased disease activity according to DAS28.

### Experienced RA’s influence on my everyday life

Looking at activity limitation through HAQ, there were no statistically significant differences between men and women. However, men displayed more activity limitation than women upon inclusion (men mean score 0.9, 95% CI = 0.6 to 1.2; women mean score 0.7, 95% CI = 0.4 to 1.0) and 10 years (men mean score 0.5, 95% CI = 0.2 to 0.7; women mean score 0.4, 95% CI = 0.2 to 0.7), whereas women’s HAQ score was higher than men during the years in between (Fig. 3). Compared to the results from the qualitative analysis, the integration shows *confirming* results regarding both women and men. This is since women mentioned activity limitation to a slightly larger extent three years post-diagnosis (15 mentioned problems at three, 14 mentioned problems at 10 years; HAQ mean 0.5, 95% CI = 0.3 to 0.7 at three years), and men mentioned activity limitation to a slightly larger extent 10 years after diagnosis (12 mentioned issues at three years, 13 mentioned issues at 10 years; HAQ mean score at three years 0.3, 95% CI = 0.0 to 0.5). As described in the Methods section, activity limitation related to HAQ refers to issues with activities such as walking or basic needs.

When it comes to basic needs, such as dressing and eating, a few patients disclosed issues. For instance, three years after diagnosis one man described problems in relation to his jaw, as illustrated:

*I started to feel it already if I talked or if I opened my mouth too much and then it came more and more and it felt when I ate too*,* and then it became*,* when I ate meat and chewier stuff. Yes*,* it just got worse and worse*,* and so. (Man*,* age 26*,* three years after diagnosis)*.

A decade after diagnosis, only two women expressed issues regarding basic needs. This does not completely reflect the VLA scores, however, since a larger number of patients stated basic needs as affected in the questionnaires. According to the quantitative data (not shown in Table 1), 44% of the women and 33% of the men in the sample stated their basic needs were affected 10 years after diagnosis according to VLA. Also worth noting is that 44% of women also perceived their sleeping to be affected.

Activities connected to chores around the house were reported as challenging, both in the interviews and the VLA scores. Activities such as vacuum cleaning and cooking were mentioned by women as being problematic in the interviews, which coincides with VLA scores demonstrating that 44% of women perceived these activities as affected. According to their own statement in the interviews, most women and men could pursue gardening. However, 66% of men perceived gardening as an affected activity according to the VLA questionnaires, indicating *disconfirming* results regarding this particular activity. Heavier tasks around the house, like carpentry and changing tyres, were only mentioned by men in the interviews (four mentioned it as problematic a decade after diagnosis), and from the VLA questionnaires it was unveiled that 40% of the men perceived these activities as affecting their everyday lives.

Also, specifically related to the VLA scores, physical leisure activities were considered to be the most affected activity from the whole group’s perspective (52%). Examples of this were visible in different ways across the interviews in both women and men. For example, longer walks had been shortened or switched to bicycling, and forest walks were avoided altogether. However, one woman did go hiking 10 years after diagnosis.

Patients described RA as limiting their social life, or that adaptations were necessary. This occurred in both women and men, and during both interview sets (social life described as affected by in total 10 patients at three years and, by 10 patients at 10 years). A decade after diagnosis, VLA scores displayed that 38% of both women and men perceived their possibility to social participation as being affected, illustrating *confirming* results in the integration phase. The following quote exemplifies RA’s impact on social activities:

*I mean it is limited. So*,* I*,* if I am to plan something that I am supposed to do*,* then I do that on a day when I am off if possible and sometimes I withdraw from things because I can not bear it if I have been working from nine to four*,* so maybe I do not have the energy to do anything in the evening. (Woman*,* age 61*,* 10 years after diagnosis)*

Other aspects of everyday life were the fact that activities took longer time to perform and necessary adaptation that followed. For example, patients could experience a lack of energy, feel forced to take breaks during activities or split the activities across several days. On the one hand, everyday life was considered to be changed, and on the other, activities could still be performed but over a longer time frame.

At both three and 10 years, both male and female patients expressed how RA required them to make adaptations in their everyday lives (described by totally 16 at three years, totally 15 at 10 years). This could include adapting one’s planning day-by-day but could also mean avoiding activities altogether. A general feeling of needing to adapt was expressed, as illustrated in the following examples:

*Most of the things can be done*,* but it may take the time it needs. (Man*,* age 44*,* three years after diagnosis)*

*Well*,* I do not live like I did before when I was healthy*,* it is really a difference. (Woman*,* age 53*,* three years after diagnosis)*

Nevertheless, in several cases, RA did not have a considerable impact on everyday life. Both men and women described how they did not need to adapt or withdraw from any activities and experienced no limitations.

*I can still do quite a lot anyway. As long as you learn to adapt. That is the way it is. (Woman*,* age 59*,* 10 years after diagnosis)*.

To summarise the second category, activity limitation according to HAQ was consistent with the patients’ experiences from the interviews. In this sense, RA’s influence on activities in everyday life displayed *confirming* results from the analyses. However, looking at the VLA scores, at 10 years after diagnosis, fewer patients expressed issues with activities such as basic needs (only three patients expressed problems) and gardening (disclosed as problematic by one patient), whereas the VLA scores in fact displayed problems to a larger extent (whole group: basic needs 39%, gardening 42%), showing *disconfirming* results. On the other hand, the VLA scores and the interviews exhibited *confirming* results regarding RA’s distinct influence on physical leisure activities and social activities.

## Discussion

The integration of quantitative and qualitative results showed both agreement and discrepancies between quantitative questionnaires and the interviews at both three and 10 years after diagnosis. It seems evident that the two perspectives complement each other in our understanding of RA’s influence on everyday life during patients’ first decade with the diagnosis. Differences between women and men over the course of the first decade after diagnosis were also displayed.

Disease activity as well as pain was overall higher in women during the first decade after RA diagnosis, which corresponds to previous research displaying differences between sexes [30, 31]. Worth noting is that all patients in our study had access to the early and improved treatment from the start of their diagnosis. Improvements in pain and activity limitation have recently been reported, for example, when comparing newer (DMARDs) and previous (methotrexate) treatment [32], however, despite decreased disease activity, women in our study still express issues with related activities. Although, symptoms such as pain are still reported by patients with RA, despite remission [4], which illustrates the chronic nature of the diagnosis and highlights the need for a continuous care and rehabilitation of good quality. Difficulties in performing valued life activities have previously been connected to higher levels of pain [30], which was seen in both men and women in our study. It is therefore important to look at the impact of pain in a wider manner, as also stated in international recommendations [33], where pain assessments should include patients’ needs in terms of priorities in everyday life. Questionnaires in combination with thorough patient interviews can contribute to this, as was performed in our study.

RA diagnosis is unquestionably associated with both activity limitations and participation restrictions. For example, in a study by Gikaro and colleagues [34], 45% of patients reported difficulties in one or more activities of daily living or participation, and nearly two thirds of patients who reported difficulties were women. Further, Leino and colleagues [35] found that most of patients report disadvantages in performing household activities, physically demanding tasks causing the most impairments, and leisure activities were reduced or abandoned. In relation to these previous research results, among our patients, physically demanding leisure activities stood out as being affected among both men and women. To some extent, household activities were also perceived as being affected, and more so among women than men.

RA’s impact on activities in everyday life was seen in our results through both a quantitative and qualitative perspective. Previous research report that patients put greater importance on symptoms’ impact on everyday life, rather than the duration or severity of the symptom; e.g., stiffness [17]. Additionally, while patient-reported outcome measures (PROMs) assess, e.g., functional limitations, they often fail to show their impact on the context of the patients’ lives [36]. This emphasises the patients’ focus on everyday life rather than on clinical outcomes. This goes hand-in-hand with disease remission, which has been defined by patients as autonomy, return to normalcy, being physically able, and having control over one’s life [37]. This definition does not coincide with clinical remission, which instead focuses on the absence of inflammatory disease activity [38]. This further points to a discrepancy between measures and the patients’ experience, something that was displayed in our study through, for example, the fact that women expressed issues with activities in everyday life in relation to symptoms like stiffness, despite a decreased disease activity a decade after diagnosis. As suggested by, for example, Acebes and colleagues [39], remission should be defined not only from the perspective of the professional, but also – and more importantly – from the patient’s perspective. Therefore, in a rehabilitation process, it is important for health care professionals to keep both clinical outcomes and the patients’ own accounts in mind.

Negrón et al. [40] highlight the influence of contextual factors on outcomes, as well as the importance of assessing patient-centred outcomes in long-term longitudinal observational studies. Our study’s contribution to this discussion are the occasionally disconfirming results indicating a need for assessments targeting different aspects. Furthermore, a patient may score ‘well’ in a disability assessment but still experience disability in more complex activities [27]. Additionally, activities such as caring for family members, driving a car, sleeping, and social activities have previously been identified as important aspects impacting everyday life, but missing from PROMs [36]. However, these activities are all visible through the VLA scale, indicating a need for a more extensive use of this assessment, and we believe it has added valuable nuances to our results.

With the aim of exploring the relationship between quantitative outcomes and patients’ subjective experiences during their first decade with RA, a mixed methods approach was considered a good fit to capture the essence of different aspects of the patients’ everyday lives. However, our sample was quantitively limited, and we dealt with missing values. Linear mixed models with restricted maximum likelihood estimation were used, as they are often preferred over more traditional approaches due to their advantages in dealing with missing values. Also, from a qualitative methodological perspective, our sample of 31 patients is a fair number of participants. Moreover, the ten-year follow-up regarding the register-based data and the second set of interviews were not performed simultaneously, presenting a limitation regarding our timeline. We have chosen to be flexible considering the interview time span, something that can, of course, affect our results. Nevertheless, after thorough discussions, this flexibility was considered reasonable in order to approach the research aim from a longitudinal perspective. However, the assessments that were used are valid and established in rheumatic diseases. Finally, our patients were recruited from a well-managed and controlled cohort, which adds validity to our study.

## Conclusion

Our integration of quantitative and qualitative data shows that many patients in our study still exhibit disability a decade after diagnosis, despite access to modern treatment and decreased disease activity. Our results show that there are discrepancies between the different sets of data, namely, patients’ own experiences do not always coincide with reported outcomes. Furthermore, these discrepancies are still visible several years after diagnosis. Additionally, patients’ experiences of disability add essential input to quantitative data. Our results confirm the importance of thorough interviews in clinical settings, alongside valid assessments of disability, to design person-centered rehabilitation to optimise the patients’ possibilities for participation in everyday life over a long period of time.

## Electronic Supplementary Material

Below is the link to the electronic supplementary material.

Supplementary Material 1

Supplementary Material 2

Supplementary Material 3

## Data Availability

The datasets generated and analysed during the current study are not publicly available due to the nature of ethical approval, but anonymised data are available from the corresponding author on reasonable request.

## Abbreviations

ACR87: American College of Rheumatology classification criteria of RA
DAS28: Disease Activity Score in 28 joints
DMARD: Disease-modifying antirheumatic drug
HAQ: Health Assessment Questionnaire
PROM: Patient-reported outcome measure
RA: Rheumatoid arthritis
SRQ: The Swedish Rheumatology Quality Register
TIRA-2: Early Interventions in Rheumatoid Arthritis
VAS: Visual Analogue Scale
VLA-swe: Valued Life Activity Scale – Swedish version
