# Supplementary material for: “As long as you learn to adapt”–a longitudinal mixed-methods study exploring the first decade with rheumatoid arthritis
Source: BMC Rheumatol. 2025 Mar 24;9:35. doi: 10.1186/s41927-025-00485-z (PMC11931753; doi:10.1186/s41927-025-00485-z)
Supplement: Supplementary file 1 — Supplementary Material 1 [file 41927_2025_485_MOESM1_ESM.pdf]

Supplementary file 1

Interview guide 3 years after diagnosis

1. How well do you feel that your everyday life works?

2. Can you share an experience (from the past week, if possible) when your rheumatism limited you in everyday life?

When? Explain: What was the incident/situation? Who else was there?

Did this event/incident have any consequences for you? Explain:
What happened, what were you thinking, what did you feel?

How did you handle this incident/situation?

Did you feel you were able to influence this situation? Own power/influence

3a. Do you feel that you give particular consideration to your rheumatism in everyday life?
3b. Do you feel that you give particular consideration to your rheumatism in your interactions with other people?

4. Can you share an experience (from the past week, if possible) when you gave particular consideration to your rheumatism in your interactions with other people?

When? Explain: What was the incident/situation? Who else was there?

Did this event/incident have any consequences for you? Explain:
What happened, what were you thinking, what did you feel?

How did you handle this incident/situation?

Did you feel you were able to influence this situation? Own power/influence

5a. Can you share additional situations (from the past week, if possible) when you adjusted your behaviour in your interactions with other people because of your rheumatism?
Skip to question 5a unless a specific situation can be found, ask instead.

5b. How does your daily life function, what does a day out look like for you, what do you do, describe a day, at home, at work and on your own time?

Continue 5a. When? Explain: What was the incident/situation? Who else was there?

Did this event/incident have any consequences for you? Explain:
What happened, what were you thinking, what did you feel?

How did you handle this incident/situation?

Did you feel you were able to influence this situation? Own power/influence

6. What do you think of when I say the word participation?

7a. Can you explain/describe how you experience participation?
7b. When do you experience participation with others?

8. Can you tell me about a time when you experienced participation in interactions with others; preferably from the past week.

When? Explain: What was the incident/situation? Who else was there?

Did this event/incident have any consequences for you? Explain:
What happened, what were you thinking, what did you feel?

How did you handle this incident/situation?

Did you feel you were able to influence this situation? Own power/influence

9. Can you tell me about another time when you experience participation in interactions with others; preferably from the past week.
Skip the rest of question 9 unless a special situation is mentioned. If no answer given about participation, ask about a situation in which the person felt a lack of participation, then proceed with the situation description.

9b. Do you sometimes feel excluded or alienated because of RA?

When? Explain: What was the incident/situation? Who else was there?

Did this event/incident have any consequences for you? Explain:
What happened, what were you thinking, what did you feel?

How did you handle this incident/situation?

Did you feel you were able to influence this situation? Own power/influence

10. What do you feel is the relevance of being a man or woman to the experiences you described above and in relation to your RA?

11. Do you think having RA has an impact on your intimate and close relations (sexual)? If so, please share with us in what way it impacts these relations?

12. Is there anything more that I have not asked but that you would like to share with us?
