# Supplementary material for: “As long as you learn to adapt”–a longitudinal mixed-methods study exploring the first decade with rheumatoid arthritis
Source: BMC Rheumatol. 2025 Mar 24;9:35. doi: 10.1186/s41927-025-00485-z (PMC11931753; doi:10.1186/s41927-025-00485-z)
Supplement: Supplementary file 2 — Supplementary Material 2 [file 41927_2025_485_MOESM2_ESM.pdf]

Supplementary file 2

Interview guide, 10 years after diagnosis

- Please, describe an ordinary day

- Do you feel that you give particular consideration to your rheumatism in everyday life?

  Can you share an experience when you gave particular consideration to your rheumatism in everyday life?

  What consequences did this situation have for you?
  What were you thinking and what did you feel in that situation?

- What do you think of when I say the word participation?

  When do you experience participation in everyday life?
  When do you experience participation with others?

- Do you feel that you give particular consideration to your rheumatism in your interactions with other people?

  Can you share an experience when you gave particular consideration to your rheumatism in your interactions with other people?

- What does support mean to you?

  Can you describe how you feel supported in participating in your everyday life?
  Do you consider your significant other to be a source of support? In that case, what kind of support?
  How do you experience that support?

- Do you consider yourself needing additional support to feel even greater participation in your everyday life?

  What kind of support?

- How do you reflect on the fact that you and your significant other can support each other?

- Do you feel that the support you receive and your need for support have changed over time, and if so, in what way?

- Is there something I have not asked you that you would like to share? Something in your life related to rheumatism? Something related to your relationship with your significant other?

<u>Our definition of participation</u>
What you do and what you feel is important, that engages you, when you feel a sense of belonging, power, and impact, preferably in collaboration with others.
