# Supplementary material for: “As long as you learn to adapt”–a longitudinal mixed-methods study exploring the first decade with rheumatoid arthritis
Source: BMC Rheumatol. 2025 Mar 24;9:35. doi: 10.1186/s41927-025-00485-z (PMC11931753; doi:10.1186/s41927-025-00485-z)
Supplement: Supplementary file 3 — Supplementary Material 3 [file 41927_2025_485_MOESM3_ESM.pdf]

Linear Mixed Model on DAS28 with timepoint, sex, and their interaction as fixed effects

Mixed Model Analysis

Case Processing Summary

		Label	Count	Marginal Percentage
ID	1		4	3,2%
	2		3	2,4%
	3		3	2,4%
	4		4	3,2%
	5		5	4,0%
	6		4	3,2%
	7		3	2,4%
	8		5	4,0%
	9		4	3,2%
	10		5	4,0%
	11		4	3,2%
	12		4	3,2%
	13		4	3,2%
	14		3	2,4%
	15		4	3,2%
	16		5	4,0%
	17		4	3,2%
	18		4	3,2%
	19		5	4,0%
	20		4	3,2%
	21		5	4,0%
	22		5	4,0%
	23		3	2,4%
	24		4	3,2%
	25		3	2,4%
	26		4	3,2%
	27		5	4,0%
	28		3	2,4%
	29		3	2,4%
	30		5	4,0%
	31		4	3,2%
Timepoint	1	BL	29	23,2%
	2	3Y	27	21,6%
	3	5Y	28	22,4%
	4	8Y	24	19,2%
	5	10Y	17	13,6%
Sex	0	Female	68	54,4%
	1	Male	57	45,6%
Valid			125	100,0%
Excluded			30	
Total			155	

Model Dimension[a]

		Number of Levels	Covariance Structure	Number of Parameters	Subject Variables	Number of Subjects
Fixed Effects	Intercept	1		1		
	Timepoint	5		4		
	Sex	2		1		
	Timepoint * Sex	10		4		
Repeated Effects	Timepoint	5	Unstructure d	15	ID	31
Total		23		25		

a. Dependent Variable: Disease Activity Score (DAS-28).

Linear Mixed Model on DAS28 with timpoint, sex, and their interaction as fixed effects

Information Criteria[a]

-2 Restricted Log Likelihood	374,400
Akaike's Information Criterion (AIC)	404,400
Hurvich and Tsai's Criterion (AICC)	409,248
Bozdogan's Criterion (CAIC)	460,574
Schwarz's Bayesian Criterion (BIC)	445,574

The information criteria are displayed in smaller-is-better form.

a. Dependent Variable: Disease Activity Score (DAS-28).

Fixed Effects

Type III Tests of Fixed Effects[a]

Source	Numerator df	Denominator df	F	Sig.
Intercept	1	29,170	429,196	<,001
Timepoint	4	24,089	22,843	<,001
Sex	1	29,170	2,420	,131
Timepoint * Sex	4	24,089	,476	,753

a. Dependent Variable: Disease Activity Score (DAS-28).

Estimates of Fixed Effects[a]

Parameter	Estimate	Std. Error	df	t	Sig.	95% Confidence Interval Lower Bound	95% Confidence Interval Upper Bound
Intercept	2,636128	,362842	18,687	7,265	<,001	1,875828	3,396428
[Timepoint=1]	1,647338	,573326	24,722	2,873	,008	,465878	2,828799
[Timepoint=2]	-,607013	,441417	21,586	-1,375	,183	-1,523473	,309447
[Timepoint=3]	-,319162	,436691	27,121	-,731	,471	-1,214992	,576668
[Timepoint=4]	-,268605	,383104	21,989	-,701	,491	-1,063137	,525926
[Timepoint=5]	0[b]	0	.	.	.	.	.
[Sex=0]	-,020910	,498332	18,555	-,042	,967	-1,065628	1,023808
[Sex=1]	0[b]	0	.	.	.	.	.
[Timepoint=1] * [Sex=0]	,869944	,792266	23,932	1,098	,283	-,765457	2,505345
[Timepoint=1] * [Sex=1]	0[b]	0	.	.	.	.	.
[Timepoint=2] * [Sex=0]	,761584	,601031	21,255	1,267	,219	-,487416	2,010583
[Timepoint=2] * [Sex=1]	0[b]	0	.	.	.	.	.
[Timepoint=3] * [Sex=0]	,228319	,574500	25,110	,397	,694	-,954624	1,411262
[Timepoint=3] * [Sex=1]	0[b]	0	.	.	.	.	.
[Timepoint=4] * [Sex=0]	,458513	,525581	21,449	,872	,393	-,633101	1,550127
[Timepoint=4] * [Sex=1]	0[b]	0	.	.	.	.	.
[Timepoint=5] * [Sex=0]	0[b]	0	.	.	.	.	.
[Timepoint=5] * [Sex=1]	0[b]	0	.	.	.	.	.

a. Dependent Variable: Disease Activity Score (DAS-28).

b. This parameter is set to zero because it is redundant.

Linear Mixed Model on DAS28 with timpoint, sex, and their interaction as fixed effects

Covariance Parameters

Estimates of Covariance Parameters[a]

Parameter		Estimate	Std. Error	Wald Z	Sig.	95% Confidence Interval Lower Bound	Upper Bound
Repeated Measures	UN (1,1)	1,710166	,500195	3,419	<,001	,963999	3,033893
	UN (2,1)	,353797	,278063	1,272	,203	-,191196	,898790
	UN (2,2)	1,058529	,313749	3,374	<,001	,592114	1,892343
	UN (3,1)	,184619	,401184	,460	,645	-,601686	,970925
	UN (3,2)	,832941	,368842	2,258	,024	,110023	1,555858
	UN (3,3)	2,426930	,661156	3,671	<,001	1,422882	4,139479
	UN (4,1)	,306986	,299254	1,026	,305	-,279541	,893512
	UN (4,2)	,264827	,235667	1,124	,261	-,197072	,726725
	UN (4,3)	,342069	,342501	,999	,318	-,329220	1,013358
	UN (4,4)	1,132049	,333324	3,396	<,001	,635674	2,016023
	UN (5,1)	-,603324	,367036	-1,644	,100	-1,322701	,116053
	UN (5,2)	,147921	,263650	,561	,575	-,368823	,664665
	UN (5,3)	1,032101	,478644	2,156	,031	,093975	1,970226
	UN (5,4)	,517851	,305806	1,693	,090	-,081518	1,117221
	UN (5,5)	1,439022	,552568	2,604	,009	,677978	3,054352

a. Dependent Variable: Disease Activity Score (DAS-28).

Custom Hypothesis Test 1 (Within-subject change 3Y - BL, [L1] Female, [L2] Male)

Contrast Estimates[a,b]

Contrast	Estimate	Std. Error	df	Test Value	t	Sig.	95% Confidence Interval Lower Bound	Upper Bound
L1	-2,362711	,362831	24,162	0	-6,512	<,001	-3,111292	-1,614130
L2	-2,254351	,400717	28,829	0	-5,626	<,001	-3,074121	-1,434581

a. Within-subject change 3Y - BL, [L1] Female, [L2] Male

b. Dependent Variable: Disease Activity Score (DAS-28).

Test of Contrasts[a]

Source	Numerator df	Denominator df	F	Sig.
Within-subject change 3Y - BL, [L1] Female, [L2] Male	2	26,273	37,027	<,001

a. Dependent Variable: Disease Activity Score (DAS-28).

Custom Hypothesis Test 2 (Within-subject change 5Y - BL, [L1] Female, [L2] Male)

Contrast Estimates[a,b]

Contrast	Estimate	Std. Error	df	Test Value	t	Sig.	95% Confidence Interval Lower Bound	Upper Bound
L1	-2,608125	,485274	25,257	0	-5,375	<,001	-3,607050	-1,609200
L2	-1,966500	,529139	29,224	0	-3,716	<,001	-3,048351	-,884649

a. Within-subject change 5Y - BL, [L1] Female, [L2] Male

b. Dependent Variable: Disease Activity Score (DAS-28).

Test of Contrasts[a]

Source	Numerator df	Denominator df	F	Sig.
Within-subject change 5Y - BL, [L1] Female, [L2] Male	2	27,085	21,349	<,001

a. Dependent Variable: Disease Activity Score (DAS-28).

Linear Mixed Model on DAS28 with timpoint, sex, and their interaction as fixed effects

Custom Hypothesis Test 3 (Within-subject change 8Y - BL, [L1] Female, [L2] Male)

Contrast Estimates[a,b]

Contrast	Estimate	Std. Error	df	Test Value	t	Sig.	95% Confidence Interval Lower Bound	95% Confidence Interval Upper Bound
L1	-2,327374	,398041	28,218	0	-5,847	<,001	-3,142440	-1,512309
L2	-1,915944	,419622	30,014	0	-4,566	<,001	-2,772910	-1,058978

a. Within-subject change 8Y - BL, [L1] Female, [L2] Male

b. Dependent Variable: Disease Activity Score (DAS-28).

Test of Contrasts[a]

Source	Numerator df	Denominator df	F	Sig.
Within-subject change 8Y - BL, [L1] Female, [L2] Male	2	29,087	27,518	<,001

a. Dependent Variable: Disease Activity Score (DAS-28).

Custom Hypothesis Test 4 (Within-subject change 10Y - BL, [L1] Female, [L2] Male)

Contrast Estimates[a,b]

Contrast	Estimate	Std. Error	df	Test Value	t	Sig.	95% Confidence Interval Lower Bound	95% Confidence Interval Upper Bound
L1	-2,517282	,546792	23,082	0	-4,604	<,001	-3,648185	-1,386379
L2	-1,647338	,573326	24,722	0	-2,873	,008	-2,828799	-,465878

a. Within-subject change 10Y - BL, [L1] Female, [L2] Male

b. Dependent Variable: Disease Activity Score (DAS-28).

Test of Contrasts[a]

Source	Numerator df	Denominator df	F	Sig.
Within-subject change 10Y - BL, [L1] Female, [L2] Male	2	23,872	14,725	<,001

a. Dependent Variable: Disease Activity Score (DAS-28).

Custom Hypothesis Test 5 (Between-subject effects Female - Male, [L1] 3Y - BL, [L2] 5Y - BL, [L3] 8Y - BL, [L4] 10Y - BL)

Contrast Estimates[a,b]

Contrast	Estimate	Std. Error	df	Test Value	t	Sig.	95% Confidence Interval Lower Bound	95% Confidence Interval Upper Bound
L1	-,108360	,540575	26,640	0	-,200	,843	-1,218230	1,001510
L2	-,641625	,717969	27,421	0	-,894	,379	-2,113717	,830467
L3	-,411431	,578377	29,189	0	-,711	,483	-1,594010	,771149
L4	-,869944	,792266	23,932	0	-1,098	,283	-2,505345	,765457

a. Between-subject effects Female - Male, [L1] 3Y - BL, [L2] 5Y - BL, [L3] 8Y - BL, [L4] 10Y - BL

b. Dependent Variable: Disease Activity Score (DAS-28).

Test of Contrasts[a]

Source	Numerator df	Denominator df	F	Sig.
Between-subject effects Female - Male, [L1] 3Y - BL, [L2] 5Y - BL, [L3] 8Y - BL, [L4] 10Y - BL	4	21,566	,476	,753

a. Dependent Variable: Disease Activity Score (DAS-28).

Linear Mixed Model on DAS28 with timpoint, sex, and their interaction as fixed effects

Estimated Marginal Means

1. Timepoint

Estimates[a]

Timepoint	Mean	Std. Error	df	95% Confidence Interval Lower Bound	95% Confidence Interval Upper Bound
BL	4,708	,241	24,498	4,212	5,204
3Y	2,399	,195	24,474	1,998	2,801
5Y	2,421	,288	28,481	1,830	3,011
8Y	2,586	,212	24,086	2,148	3,025
10Y	2,626	,249	18,555	2,103	3,148

a. Dependent Variable: Disease Activity Score (DAS-28).

Pairwise Comparisons[a]

(I) Timepoint	(J) Timepoint	Mean Difference (I-J)	Std. Error	df	Sig.[c]	95% Confidence Interval for Difference[c] Lower Bound	95% Confidence Interval for Difference[c] Upper Bound
3Y	BL	-2,309*	,270	26,640	<,001	-3,033	-1,584
5Y	BL	-2,287*	,359	27,421	<,001	-3,247	-1,328
8Y	BL	-2,122*	,289	29,189	<,001	-2,891	-1,352
10Y	BL	-2,082*	,396	23,932	<,001	-3,152	-1,012

Based on estimated marginal means

*. The mean difference is significant at the ,05 level.

a. Dependent Variable: Disease Activity Score (DAS-28).

c. Adjustment for multiple comparisons: Bonferroni.

Univariate Tests[a]

Numerator df	Denominator df	F	Sig.
4	21,566	22,843	<,001

The F tests the effect of Timepoint. This test is based on the linearly independent pairwise comparisons among the estimated marginal means.

a. Dependent Variable: Disease Activity Score (DAS-28).

2. Sex

Estimates[a]

Sex	Mean	Std. Error	df	95% Confidence Interval	
				Lower Bound	Upper Bound
Female	3,169	,197	28,319	2,766	3,573
Male	2,727	,205	29,958	2,307	3,146

a. Dependent Variable: Disease Activity Score (DAS-28).

Pairwise Comparisons[a]

(I) Sex	(J) Sex	Mean Difference (I-J)	Std. Error	df	Sig.[b]	95% Confidence Interval for Difference[b]	
						Lower Bound	Upper Bound
Female	Male	,443	,285	29,170	,131	-,139	1,025
Male	Female	-,443	,285	29,170	,131	-1,025	,139

Based on estimated marginal means

a. Dependent Variable: Disease Activity Score (DAS-28).

b. Adjustment for multiple comparisons: Least Significant Difference (equivalent to no adjustments).

Univariate Tests[a]

Numerator df	Denominator df	F	Sig.
1	29,170	2,420	,131

The F tests the effect of Sex. This test is based on the linearly independent pairwise comparisons among the estimated marginal means.

a. Dependent Variable: Disease Activity Score (DAS-28).

3. Timepoint * Sex

Estimates[a]

Timepoint	Sex	Mean	Std. Error	df	95% Confidence Interval Lower Bound	95% Confidence Interval Upper Bound
BL	Female	5,133	,327	23,379	4,457	5,808
	Male	4,283	,353	25,432	3,556	5,010
3Y	Female	2,770	,263	23,569	2,227	3,312
	Male	2,029	,287	25,203	1,438	2,620
5Y	Female	2,524	,389	26,949	1,725	3,324
	Male	2,317	,426	29,533	1,447	3,187
8Y	Female	2,805	,300	24,145	2,186	3,424
	Male	2,368	,301	24,021	1,747	2,988
10Y	Female	2,615	,342	18,385	1,899	3,332
	Male	2,636	,363	18,687	1,876	3,396

a. Dependent Variable: Disease Activity Score (DAS-28).

Pairwise Comparisons[a]

Timepoint	(I) Sex	(J) Sex	Mean Difference (I-J)	Std. Error	df	Sig.[b]	95% Confidence Interval for Difference[b] Lower Bound	95% Confidence Interval for Difference[b] Upper Bound
BL	Female	Male	,849	,481	24,498	,090	-,143	1,841
	Male	Female	-,849	,481	24,498	,090	-1,841	,143
3Y	Female	Male	,741	,389	24,474	,069	-,062	1,543
	Male	Female	-,741	,389	24,474	,069	-1,543	,062
5Y	Female	Male	,207	,577	28,481	,722	-,973	1,388
	Male	Female	-,207	,577	28,481	,722	-1,388	,973
8Y	Female	Male	,438	,425	24,086	,313	-,439	1,314
	Male	Female	-,438	,425	24,086	,313	-1,314	,439
10Y	Female	Male	-,021	,498	18,555	,967	-1,066	1,024
	Male	Female	,021	,498	18,555	,967	-1,024	1,066

Based on estimated marginal means

a. Dependent Variable: Disease Activity Score (DAS-28).

b. Adjustment for multiple comparisons: Least Significant Difference (equivalent to no adjustments).

Univariate Tests[a]

Timepoint	Numerator df	Denominator df	F	Sig.
BL	1	24,498	3,111	,090
3Y	1	24,474	3,623	,069
5Y	1	28,481	,129	,722
8Y	1	24,086	1,061	,313
10Y	1	18,555	,002	,967

Each F tests the simple effects of Sex within each level combination of the other effects shown. These tests are based on the linearly independent pairwise comparisons among the estimated marginal means.

a. Dependent Variable: Disease Activity Score (DAS-28).

4. Sex * Timepoint

Estimates[a]

Sex	Timepoint	Mean	Std. Error	df	95% Confidence Interval	
					Lower Bound	Upper Bound
Female	BL	5,133	,327	23,379	4,457	5,808
	3Y	2,770	,263	23,569	2,227	3,312
	5Y	2,524	,389	26,949	1,725	3,324
	8Y	2,805	,300	24,145	2,186	3,424
	10Y	2,615	,342	18,385	1,899	3,332
Male	BL	4,283	,353	25,432	3,556	5,010
	3Y	2,029	,287	25,203	1,438	2,620
	5Y	2,317	,426	29,533	1,447	3,187
	8Y	2,368	,301	24,021	1,747	2,988
	10Y	2,636	,363	18,687	1,876	3,396

a. Dependent Variable: Disease Activity Score (DAS-28).

Pairwise Comparisons[a]

Sex	(I) Timepoint	(J) Timepoint	Mean Difference (I-J)	Std. Error	df	Sig.[c]	95% Confidence Interval for Difference[c]	
							Lower Bound	Upper Bound
Female	3Y	BL	-2,363*	,363	24,162	<,001	-3,342	-1,384
	5Y	BL	-2,608*	,485	25,257	<,001	-3,913	-1,303
	8Y	BL	-2,327*	,398	28,218	<,001	-3,389	-1,265
	10Y	BL	-2,517*	,547	23,082	<,001	-3,998	-1,036
Male	3Y	BL	-2,254*	,401	28,829	<,001	-3,322	-1,187
	5Y	BL	-1,967*	,529	29,224	,003	-3,375	-,558
	8Y	BL	-1,916*	,420	30,014	<,001	-3,031	-,801
	10Y	BL	-1,647*	,573	24,722	,033	-3,192	-,103

Based on estimated marginal means

*. The mean difference is significant at the ,05 level.

a. Dependent Variable: Disease Activity Score (DAS-28).

c. Adjustment for multiple comparisons: Bonferroni.

Univariate Tests[a]

Sex	Numerator df	Denominator df	F	Sig.
Female	4	17,704	13,627	<,001
Male	4	24,701	9,952	<,001

Each F tests the simple effects of Timepoint within each level combination of the other effects shown. These tests are based on the linearly independent pairwise comparisons among the estimated marginal means.

a. Dependent Variable: Disease Activity Score (DAS-28).
